# Communicating the threat of emerging infections to the public.

**DOI:** 10.3201/eid0604.000403

**Published:** 2000

**Authors:** V Freimuth, H W Linnan, P Potter

**Affiliations:** Centers for Disease Control and Prevention, Atlanta, Georgia 30333, USA.

## Abstract

Communication theory and techniques, aided by the electronic revolution, provide new opportunities and challenges for the effective transfer of laboratory, epidemiologic, surveillance, and other public health data to the public who funds them. We review the applicability of communication theory, particularly the audience-source-message-channel meta-model, to emerging infectious disease issues. Emergence of new infectious organisms, microbial resistance to therapeutic drugs, and increased emphasis on prevention have expanded the role of communication as a vital component of public health practice. In the absence of cure, as in AIDS and many other public health problems, an effectively crafted and disseminated prevention message is the key control measure. Applying communication theory to disease prevention messages can increase the effectiveness of the messages and improve public health.


### Communicating the Threat of Emerging Infections to the Public

**Vicki Freimuth, Huan W. Linnan, and Polyxeni Potter**
Centers for Disease Control and Prevention, Atlanta, Georgia, USA

Communication theory and techniques, aided by the electronic revolution, provide new opportunities and challenges for the effective transfer of laboratory, epidemiologic, surveillance, and other public health data to the public who funds them. We review the applicability of communication theory, particularly the audience-source-message-channel meta-model, to emerging infectious disease issues. Emergence of new infectious organisms, microbial resistance to therapeutic drugs, and increased emphasis on prevention have expanded the role of communication as a vital component of public health practice. In the absence of cure, as in AIDS and many other public health problems, an effectively crafted and disseminated prevention message is the key control measure. Applying communication theory to disease prevention messages can increase the effectiveness of the messages and improve public health.

Although prevention has historically been part of medical intervention, communicating the threat of disease to the public has not always been in the forefront of public health efforts. Federal agencies have focused on the scientific accuracy of the laboratory research, disease surveillance, epidemiologic studies, and immunization efforts of their organizations. As scientific studies meticulously recorded the results of public health efforts in medical journals, reaching the public directly was, for the most part, left to the press and others who translated scientific journal information for the lay person. However, the era of AIDS, the emergence of new infectious organisms, microbial resistance to therapeutic drugs, and a new emphasis on prevention have expanded the role of communication as an important and necessary component of public health practice. At the same time, public interest in health information has soared (Figure 1) as the baby boom generation has aged and electronic publishing has broadened the audience and scope of the published word. At the crossroads of AIDS and the information age is the science of health communication, the means to disease prevention through behavior modification. In this article, we describe how communica-

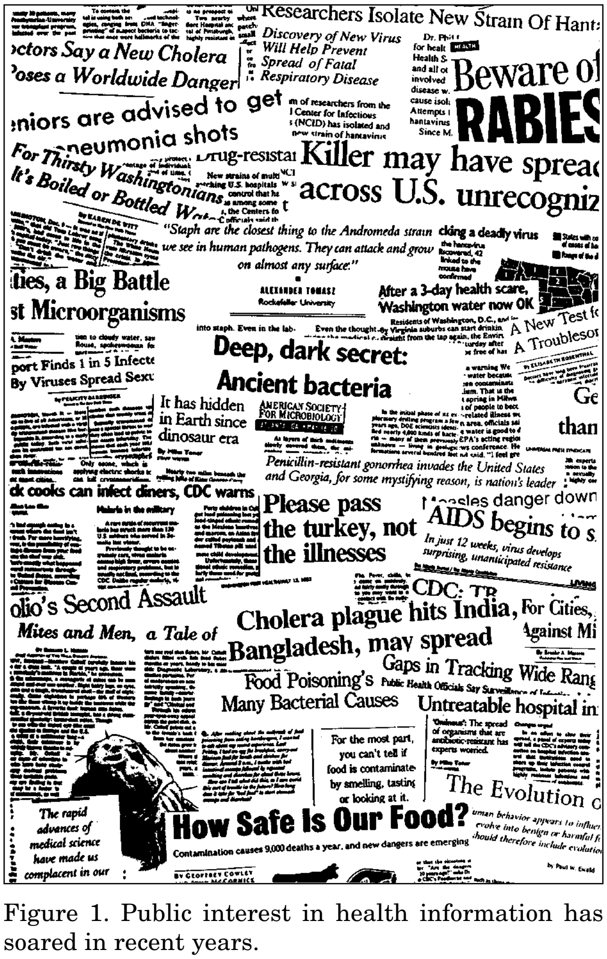

Figure 1. Public interest in health information has soared in recent years.

Address for correspondence: Vicki Freimuth, Centers for Disease Control and Prevention, 1600 Clifton Road, Mail Stop D42, Atlanta, GA 30333, USA; Fax 404-639-7391; e-mail:

tion theory can be applied to the effective prevention and control of emerging infectious diseases.

### Health Communication Methods

Health communication has been defined as the study and use of methods to inform and influence individual and community decisions that enhance health (1). Communication methods are used to create and increase public awareness of a disease; educate the public about a disease, its causes, and treatment; change a person's or group's attitudes about a disease; change individual behavior to prevent or control a disease; advocate for policy changes in favor of disease prevention and control; and create social norms that favor healthful living. Health communication theory utilizes four key elements of the communication process: audience, message, source, and channel. Effective health communication programs identify and prioritize audience segments; deliver accurate, scientifically based messages from credible sources; and reach audiences through familiar channels.

### The Communication Process

#### Audience

Understanding the audience for which a message is intended is critical to the communication process. The clearer the understanding of the audience for which a message is intended, the better the chance of developing an effective message. The audience can be divided into smaller subgroups or segments of similar internal composition. Audience segmentation allows for more specific and individually tailored messages for each subgroup. An audience can be segmented on the basis of any number of criteria: demographics (sex, age, education); behavior (outdoors activities, food-handling practices, handwashing); and psychographic characteristics (values, attitudes, life-styles).

Once an audience is segmented, the subgroups are carefully assessed (through focus groups, in-depth interviews, demographic and other data) so that appropriate messages, sources, and channels can be formulated, first to inform the subgroup populations about a disease problem and then to propose acceptable behavior changes to prevent disease and promote healthful living. A specific example can clarify the segmentation process: campers, hikers, outdoor workers, and others who frequent wooded, brushy, and grassy places can be exposed to ticks involved in the transmission of Lyme disease. Suburban residents whose homes encroach on the habitats of deer and other animals infested by ticks are also at risk. Through audience segmentation, a message intended to increase awareness of Lyme disease can be directed at youth or middle-age, middle-income suburban men and women who frequently hike or camp. This subgroup, which is likely to read materials relevant to outdoors activities, could be reached through a brief leaflet (distributed at schools or physicians' offices) describing Lyme disease and simple ways to avoid it; through Internet resources on outdoors activities, where they may peruse the geographic areas likely to be infested with Lyme disease ticks and thus assess their personal risk; and through specialty shops, where along with insect repellent and hiking and camping gear, they may see posters urging them to check for ticks and may find light-colored clothes expressly marketed for ease of spotting ticks.

Audiences can also be segmented according to Prochaska's Stages of Change model (2), which suggests that behavior changes slowly, through a sequence of stages: precontemplation, contemplation, preparation, action, and maintenance. For example, the message, source, and channel may be different for an audience that has just heard of Lyme disease and is thinking about how likely it is to affect them (contemplation stage) than for an audience that has actively begun to take precautions (action stage) but needs reinforcement to make that behavior an integral part of their activities. Figure 2 portrays someone in the action stage who needs help integrating the message into normal life activities.

#### Message

Effective health communication messages follow some general principles (3): they are clear and simple, positive, and both emotional and rational; if they arouse fear, they show ways of alleviating the fear; and if they contain motivational appeals, the appeals follow established guidelines likely to produce the expected response.

On the individual or intrapersonal level, effective health communication messages often apply Prochaska's Stages of Change model and the Health Belief model to message design. The

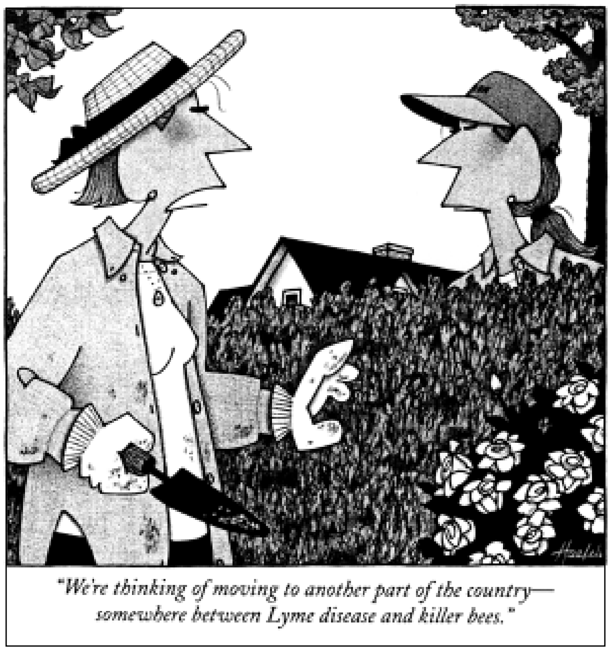

Figure 2. Disease prevention messages are slowly integrated into life activities.
(©The New Yorker Collection 1999 William Haefeli from cartoonbank.com. All Rights Reserved.)

Health Belief model (4) addresses one's perception of personal risk for the disease and the behavior change recommended for decreasing the risk. Key variables in this theory include one's perceptions of the severity and susceptibility of the health threat, benefits from the recommended actions, barriers to taking action, cues to action, motivations for prompt action, and confidence in one's ability to take action; the message addresses one or more of these variables. For example, customers dining at a restaurant read the small print on the menu next to an entree with raw oysters: "Oysters may pose a health risk if eaten raw." In deciding whether to eat the raw oysters, customers would weigh the pleasure gained against the risk taken (benefits vs. barriers). They would consider the likelihood (susceptibility) and seriousness (severity) of illness and their capacity to prevent it.

On the interpersonal or interactive level, Social Cognitive theory is often used (5); its basic principle is that family members, friends, coworkers, family physicians, and other health professionals can influence a person's health behavior. One learns not only through personal experience but also by observing the actions of others and the consequences of these actions. Perception of risk and confidence in one's ability to take action are again key variables. If the family cook always uses separate cutting boards for raw meat and for fresh vegetables and washes the boards well after each use and the family stays healthy, the observers adopt the cook's behavior and associate it with a positive outcome.

On the community or organizational level, the Diffusion of Innovations theory applies (6). According to this theory, new ideas, products, and social practices follow a pattern as they spread within a society. Key variables include characteristics of the innovation itself (relative advantage, compatibility, complexity, observability), communication channels, and social systems (social networks, norms, structures).

Reaching culturally diverse groups with messages vital to disease prevention and trying to convince group members to alter their behavior to safeguard their health may sometimes require tools that transcend explanatory language. Explanatory language tends to isolate and fragment, to describe one event followed by another in linear fashion. Figurative language tends to synthesize and combine; it can unite different levels of thought, feeling, and behavior into a holistic picture that gives a rounded perspective; and it draws on such unusual vehicles as culturally specific metaphors, e.g., idiomatic sayings or proverbs, stories, or songs that express aspects of folk wisdom in plain but effective terms (7). The American Red Cross, the Centers for Disease Control and Prevention (CDC), and other health organizations have turned to such figurative language to create effective prevention messages (8) (Figure 3).

An intended audience that is motivated to change behavior may lack the skills or resources to do so. In a multicultural society, certain groups may resist changing risky behavior for fear that the change may strip them of their core culture or because behavior change is too stressful (9). One way to approach culturally based resistance is the use of metaphorical methods to ease fears and create a less threatening environment. A well-placed proverb—for example, "cada cabeza es un mundo" ("each head is a world" or "each person has his or her own thoughts, dreams, and aspirations and has a right to them as a unique person")—uses the cultural framework to balance a person's responsibility to cultural traditions with the need for well-being and good health (10).

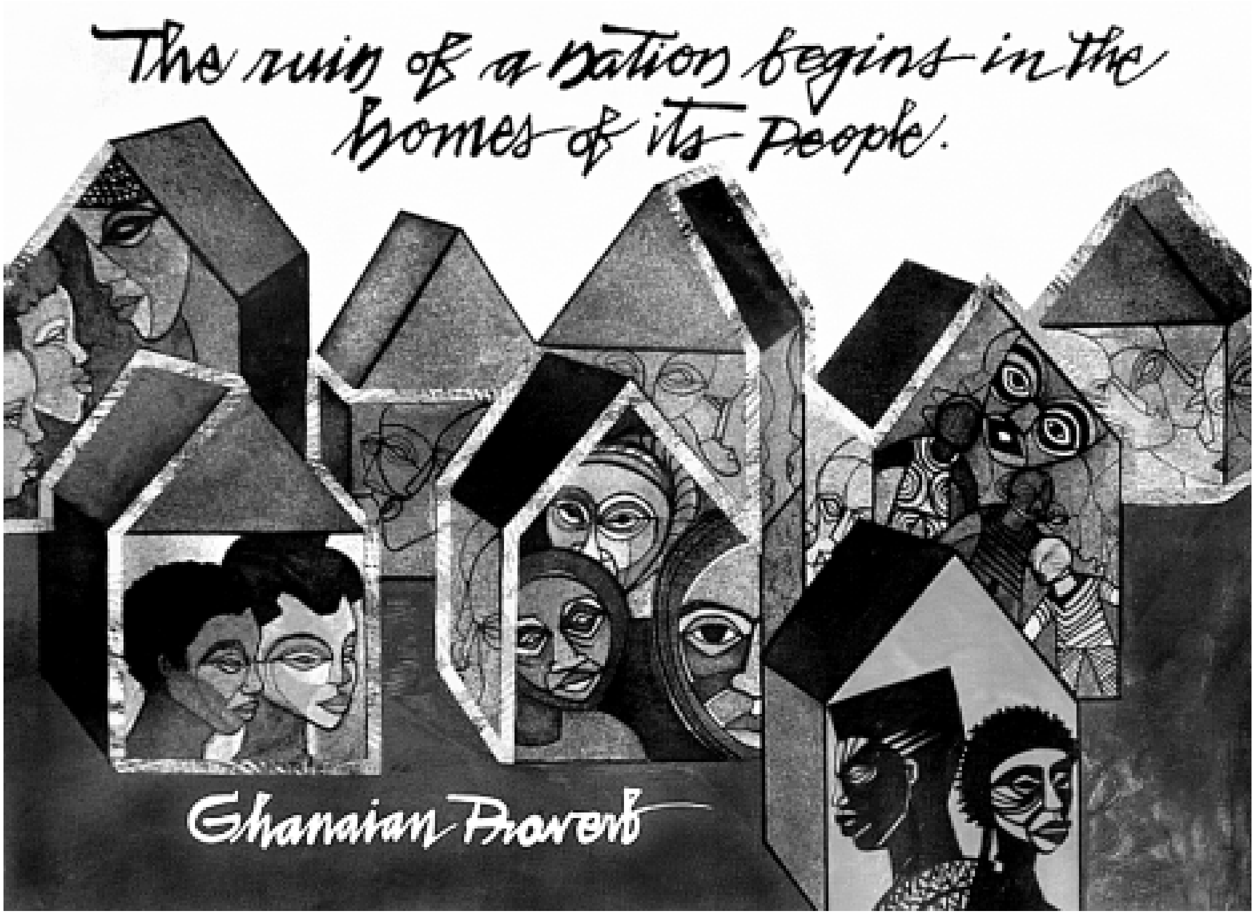

Figure 3. Illustration from the American Red Cross African-American HIV/AIDS Program poster series. Used with permission of the American Red Cross, Copyright April 1992 (revised March 1997).

Regardless of the theory and the vehicle used, the only way to know if the audience will receive the intended message is to pretest the message with a representative sample of the audience. Focus groups, personal interviews, and other techniques similar to those used in audience segmentation are ways to pretest messages. For example, focus groups conducted to explore the causes of misuse of antibiotics in pediatric practice found that physicians felt under pressure from parents to prescribe antibiotics at every office visit; on the other hand, parents indicated that they would not insist on antibiotics if the reasons for not prescribing the drugs were explained to them. Educational efforts to narrow this communication gap would reduce the unnecessary use of antibiotics and perhaps the emergence of resistant strains of *Streptococcus pneumoniae* and other pathogenic bacteria (11).

### Source

The source influences the effectiveness of the message. A source that is credible for one segment of the audience may completely miss the mark with another. While a scientist, physician, or other health-care provider may seem the ideal source of public health information, a community activist or a lay person affected by a disease may carry more credibility and have a greater public health impact. For example, a woman became a consumer activist and cofounded STOP (Safe Tables Our Priority) after her child died of hemolytic uremic syndrome, a complication of *Escherichia coli* O157:H7 infection, brought on by eating undercooked ground beef (12). The mother went on the road speaking out about her child's untimely death from eating a hamburger in a fast-food restaurant. Her moving testimonial (including poignant photographs) about the events surrounding the child's infection, illness, and death had a direct and powerful effect on the audience most likely to eat undercooked hamburgers in fast-food restaurants. The testimonial also provided an excellent springboard for discussing *E. coli* infection and individual as well as public health prevention measures. The U.S. Department of Agriculture's Pathogen Reduction and Meat and Poultry HACCP (Hazard Analysis and Critical Control Points) rule, the first major change in meat safety regulation in the United States in 90 years, was put into place in large part through the efforts of STOP, which brought parents of O157 patients to Washington, D.C., to talk to legislators about the deaths of their children from eating undercooked ground beef (J.G. Morris, pers. comm.).

### Channel

Even the best-crafted message is useless if it fails to reach the intended audience. The channel, or means by which the message is sent, is as important as the message. Mass media outlets (television, radio, magazines, newspapers, billboards, the Internet) provide ample opportunities, as do family, friends, health-care providers, and religious and other support groups (13). Other means such as telephone hot lines offer an opportunity for interpersonal communication anonymously and across geographic boundaries. Multiple channels can be combined to communicate a message more effectively. Mass media channels are most effective for increasing awareness and knowledge, but interpersonal channels work better in changing attitudes and behavior (6). A message delivered through the mass media can stimulate interpersonal discussions about a health issue. For example, a public service announcement about prevention of HIV and other sexually transmitted infections might prompt sex partners to discuss condom use (14). Computer-based communication channels (e.g., the Internet), which often provide interactive forums, are transforming and increasing channel options. By visiting various relevant web sites, a patient with chronic fatigue syndrome can locate scientific information about the disease, follow continuing research efforts, and connect with patient support groups. In 1997, during the avian influenza-like virus outbreak, the Hong Kong authorities set a new standard in communications about influenza by providing daily outbreak updates on a readily accessible Internet site. Information was also accessible on the FluNet World Health Organization Internet site (http://www.who.ch/flunet/).

### Applying Health Communication Methods

Communication research provides demographic and other information that can be used in choosing the right channel to meet the needs of specific population groups. Such communication channels include mass media campaigns, news media stories, popular entertainment, media advocacy, and interpersonal communication.

### Mass Media Campaigns

The mass media campaign, a traditional communication approach intended to produce a specific outcome within a specified period, is directed at large numbers of people through an organized set of communication activities (15). Research shows that different kinds of media affect audiences differently. For example, the news media inform and alert audiences to community developments and, in the process, shape community responses to these developments. The entertainment media fill leisure time and indirectly influence public beliefs (16,17). The business and advertising media stimulate interest in commercial goods and services and influence how and where we shop. In recent years, all these different media have also been used to disseminate health information, and for many people, they seem to have become the primary source of health information (18). The America Responds to AIDS campaign (14), which played a major role in AIDS prevention efforts, included television and radio public service announcements, printed materials (posters, booklets, brochures, billboards, bus ads), telephone hot lines, AIDS prevention messages integrated in movies and television shows, and specific AIDS information disseminated electronically through the Internet.

Mass media campaigns can raise awareness of an issue, enhance knowledge and beliefs, and reinforce existing attitudes (19-23). They can also change attitudes and behavior, especially when the change is simple and of obvious benefit to the intended audience. An intermediate objective of many mass media campaigns is to stimulate the search for additional information on a given health issue. When information-seeking is a desired outcome, mass media campaigns include a hot line for people to call for additional information. The hot line not only facilitates the search for information, it also provides a means of measuring the effect of the mass media effort. For example, a recent study found that gender, cultural values, and anxiety may affect the response of Spanish-speaking callers to HIV information over the hot lines; therefore, health educators and others who design disease prevention programs need to examine whether these programs should reinforce or challenge traditional gender roles, gender norms, or cultural values (24).

Peptic ulcer disease affects 25 million Americans and has a $6 billion impact on the nation's health-care costs. In 1994, a National Institutes of Health consensus development conference panel concluded that patients with ulcers caused by *Helicobacter pylori* infection require antibiotic treatment (25). A 1995 study showed that 72% of the general population did not know that ulcers were caused by an infection (26). The prevalent belief that ulcers were caused by stress could deter affected persons from seeking medical attention for peptic ulcer disease. Additional studies indicated that primary-care physicians were treating 50% of patients with first-time ulcer symptoms without testing for *H. pylori.* To remedy this lack of awareness of *H. pylori* infection and its relationship to peptic ulcers, Congress mandated that CDC inform consumers and health-care providers about the link between *H. pylori* infection and ulcers. CDC collaborated with other federal agencies, academic institutions, and private industry in designing and implementing an *H. pylori* educational campaign to 1) inform consumers that ulcers are caused by an infection that can be cured; 2) increase physicians' awareness of *H. pylori*, its link to ulcers, and methods for its diagnosis and treatment; and 3) improve communication between patients and health-care providers about peptic ulcer disease and the successful treatment of *H. pylori*. Focus groups (consumer, private and managed-care physicians, pharmacists) were used to determine the structure and direction of a campaign. Materials developed as a result of the focus groups included a consumer brochure, a fact sheet for health-care providers, a waiting-room poster, and television, radio, and print public service announcements. A toll-free phone number (1-888-MY-ULCER) and a web site (www.cdc.gov/ncidod/dbmd/hpylori.htm) were also established. All materials were produced in English and Spanish; copies were mailed to health-care providers and state public health agencies.

The *H. pylori* campaign was launched in October 1997 with a national media briefing in Washington, D.C. The briefing provided an overview of peptic ulcer disease and focused on diagnosis and treatment, economic impact, and research issues associated with *H. pylori.* Evaluation of media tracking records shows that extensive press coverage resulted in reaching a potential audience of more than 21 million persons since the campaign began. Qualitative research with consumers and providers is being conducted to evaluate the campaign's communication messages. Quantitative studies are ongoing to determine attitudes and behavior of ulcer patients and health-care providers.

### News Media Stories

Media stories can deliver accurate information on disease prevention in a more in-depth way than brief (paid or free) disease prevention messages (advertisements). Media stories create awareness among the intended audience, place health on the public agenda, and frame the way the issue is reported. Media stories may include the following strategies: newspaper and magazine coverage, news press conferences, press releases, video news releases, modular television or radio programming, talk show appearances, and Internet forums. Media stories are usually proactive, e.g., Parade magazine's section on "The Virus Hunters" (27) (Figure 4) and Time magazine's segment on *E. coli* infection, "The Killer Germ," (28) (Figure 5), or reactive, e.g., media coverage of the 1997 hepatitis A outbreak (150 cases) associated with frozen strawberries in Michigan (29). In the 1997 coverage, the Michigan Department of Health held a press conference announcing the outbreak and the implicated source (frozen strawberries served in public schools). Public health officials stated that 13 lots of frozen strawberries distributed to six states through the federal lunch program were possibly contaminated. Responding to the stories in the news and in the absence of information about hepatitis A, the mayor of Los Angeles held a press conference requesting 9,000 doses of immune globulin to inoculate schoolchildren who might have eaten the contaminated strawberries. Hepatitis A experts from CDC and the U.S.

Food and Drug Administration stepped in with information on hepatitis A, the proper use of vaccine, and the disease risk to the communities involved. These experts appeared on local and national news shows and delivered accurate information about the outbreak, which (having a broad geographic range and affecting children) generated extensive public interest. Through stories in the media, the public learned about hepatitis A and its causes, treatment, and prevention.

Media stories were used to alert the public about the 1993 *E. coli* O157:H7 outbreak of bloody diarrhea and serious kidney disease; the contamination (also in 1993) of a municipal water supply with the intestinal parasite *Cryptosporidium*, which caused the largest outbreak of waterborne illness in the United States; and the 1995 outbreak of Ebola hemorrhagic fever in Kikwit, Democratic Republic of Congo (then Zaire). In the Kikwit outbreak, health communication measures—which included education of physicians to recognize (or at least suspect) viral hemorrhagic fever, to reinforce the use of universal precautions and handwashing, and to collect specimens for laboratory confirmation using a method adapted to the local infrastructure—were embedded in the surveillance effort and were instrumental in stopping the outbreak and preventing further cases (30). City of Kikwit surveillance also included a mission radio network, daily voice contact by short-wave radio with 23 Catholic missions functioning in the Diocese of Kikwit, an area comprising several health zones, to inquire about possible cases and obtain follow-up information on villages with known cases within the last 3 weeks. Persons from Protestant churches and others with radios were contacted as needed. Ebola patients were visited by members of the regional surveillance team from Kikwit or by local health-care workers who had been trained in Kikwit. While being monitored for secondary cases, family members and other villagers were educated about the disease (30).

### Popular Entertainment

Popular entertainment (television shows, movies, popular songs) is effective in educating audiences about disease prevention. This strategy borrows from Albert Bandura's Social Learning theory—that most behavior is learned through modeling (31)—and involves persuading

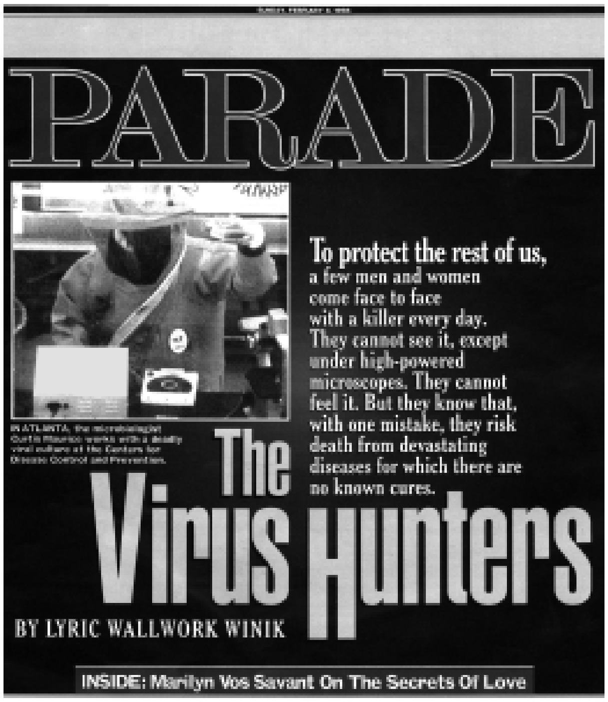

Figure 4. Cover of February 8, 1998, Parade magazine.
Used with permission of Parade Publications and Robin Thomas.

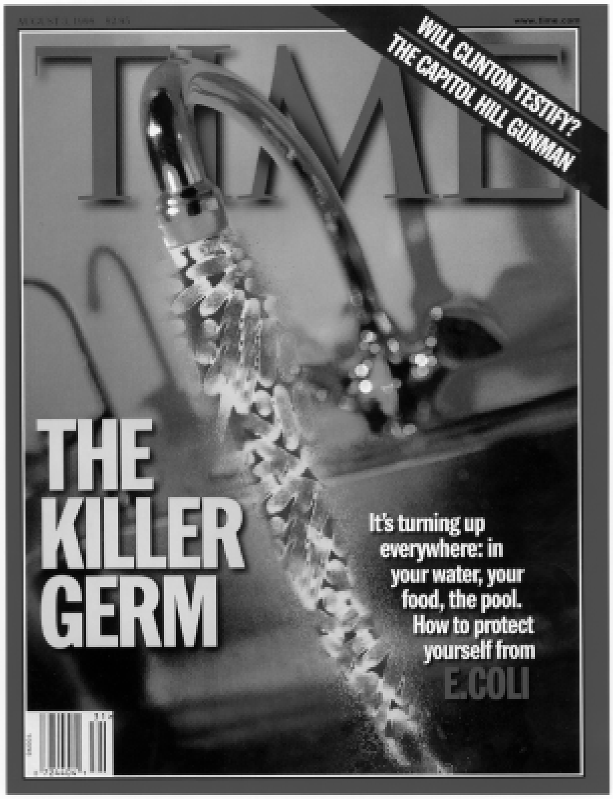

Figure 5. Cover of August 3, 1998, Time magazine (©1998 Time Inc./Timepix).

scriptwriters, directors, and producers of popular shows, movies, and other entertainment to incorporate health issues into their programs (32). The audience learns new habits and behavior by seeing them on the screen or the stage and adapting them to their own situation.

The entertainment media not only attract attention, reinforce existing behavior, and demonstrate new behavior, they also tap into the audience's emotions. When the audience responds emotionally, the educational message is more likely to influence their behavior than when they respond only rationally (33). In psychotherapy, stories are often used to motivate patients to change behavior; patients who often resist direct statements and refuse to confront complex issues directly may consider such issues if they are presented through stories (2). The television series ER, which routinely carries safe sex, blood safety, handwashing, and other health messages as part of its series, provides a safe context for messages that might otherwise be viewed as threatening or intrusive.

In Puerto Rico, Head Start centers supported by the local Rotary Club stage dramas for preschool-age children to teach them about dengue prevention, and in Sierra Leone, puppet shows and songs have been used to deliver rodent-abatement messages to local villagers to prevent Lassa fever.

### Media Advocacy

A relatively new communication approach, media advocacy, promotes changes in public health policy rather than in individual behavior (23) and stimulates media coverage to frame public debate and increase support for effective policies. Advocacy, which steers public health attention away from disease as a personal problem to health as a social issue, includes three fundamental steps: increasing media coverage and visibility of an issue, shaping the debate surrounding the issue, and advancing an effective policy (34). Media advocacy can influence public debate and put pressure on policymakers by increasing the voice of public health and visibility of values, people, and issues behind the voice (34). The goal of media advocacy is to empower the public to participate fully in defining the social and political environment in which decisions affecting health are made. This approach has been widely used in promoting the better use of antibiotics; as a result, a well-informed mother asks the pediatrician if her child's ear infection is viral or bacterial, thus relieving the physician from having to prescribe a drug that is unnecessary and ineffective. Disease information reaching the public through stories in the media during the 1997 hepatitis A outbreak in Michigan may have had broader implications for support of hepatitis A prevention programs and about policy decisions within the food industry regarding, for example, irradiation pasteurization of certain foods (35).

In the case of emerging infections, an unexpected by-product of media advocacy was the plethora of lay publications and films on infectious microbes. These microbes captured the artistic imagination of fiction writers and movie producers and brought about Outbreak, Pandora's Clock, The Hot Zone, The Coming Plague, and The Runaway Virus. In spotlighting an issue, the media also enhance the visibility, legitimacy, and power of the community. Media advocacy has played a key role in increasing awareness of emerging infections and advancing policies for their prevention and control.

In 1992, a report by the National Academy of Science's Institute of Medicine alerted the scientific community to a national (and global) infectious disease emergency: new infectious agents were emerging and old diseases thought to have been conquered were returning, often in more virulent forms; antibiotics were losing their effectiveness because infectious agents were fast becoming resistant to these drugs; and years of complacency had eroded the infectious disease infrastructure—all in an era of unprecedented travel and invasive environmental policies. This report, which might have had no impact outside the scientific community, became the center of a media campaign to raise awareness of the continued threat of infections and the need for public health action. Infectious disease experts from all over the United States drew up a strategic plan to address the threat of emerging diseases (36). Communicating the threat of these diseases and the resilient organisms that cause them was one of the plan's main goals. The plan was widely distributed to public health professionals around the world, and a summary was distributed to lawmakers. A flurry of media activity ensued. An Atlanta Journal and Constitution special segment on emerging microbes, When Bugs Fight Back (37), won the 1993 Pulitzer Prize for Explanatory Journalism.

Turner Broadcasting ran a series on infectious diseases based on J.B. McCormick's book Virus Hunters of the CDC. Media advocacy generated awareness among the public and lawmakers of the continuing threat of infectious diseases and the emergent crisis in global health.

### Interpersonal Communication

Groups or individual citizens (family members, friends, co-workers, health-care providers) can effect behavior change and stimulate community involvement through interpersonal communication (one-on-one counseling, telephone calls, interviews, community forums, training, theater, web sites). As a result of such interpersonal communication and community involvement, safe food-handling techniques and use of meat thermometers are integrated in restaurant employee training; guidelines for safe kiddy pools are posted in pool areas attended by mothers of diapered children; and water filters that can prevent cryptosporidiosis are easy to find in local hardware stores.

### Integrated Communication—Combining Strategies

Effective communication often involves multiple strategies and channels. The 1993 hantavirus outbreak in New Mexico generated an integrated communication effort involving scientific information, health-care delivery, public information to reduce panic, and dialogue between health workers and the affected Navajo community. Rapid response to the outbreak, collaboration with the affected community (including tribal medicine practitioners and elders), and sensitivity to cultural beliefs contributed to the success of the communication effort (38). Hantaviruses are rodent-borne agents spread to humans through infected rodent urine, saliva, or droppings. Campers, hikers, and others who take part in outdoors activities can become infected by breathing in the virus or (more rarely) by touching the mouth or nose after handling contaminated materials. A rodent's bite can also spread the virus.

Within hours of the first reported case of hantavirus, the Coconino County Health Department received telephone calls from the concerned public. Residents wanted to know how to recognize a deer mouse and what to do about rodents in and around their homes (38). Tourists wanted to know if it was safe to travel in and around the Four Corners area. The Coconino County Health Department, working with a public relations firm, distributed information to the city's hospitality industry. The communication link with the hospitality industry and tourists remained intact throughout the height of public concern. Because decreasing human contact with rodents was key to reducing the number of deaths, public education was critical to hantavirus prevention. Educational materials (brochures, slides, fact sheets, video tapes) in English and Navajo designed and distributed by the Indian Health Service were critical in the control of the outbreak and the prevention of additional cases (39).

Elephantiasis (lymphatic filariasis), a parasitic disease targeted for global elimination by the World Health Organization, affects 15 million people, mostly women, who become disfigured by debilitating secondary bacterial infections in the legs. The major challenge, and the key to success, in elephantiasis treatment is to convince affected women that progression of disease is not inevitable and that its outcome, to a large extent, rests in their own hands. In Brazil, acceptance of this message has led to profound and positive changes in the lives of affected women (40). In Haiti, where CDC investigators have worked on elephantiasis control for the past 10 years, the ability to effectively communicate such a message was initially limited by lack of understanding of the issues related to social stigmatization, physical ability, and barriers to changing health behavior among women with elephantiasis (41). Evaluation of patient education booklets and radio dramas broadcast locally (many developed for this project) showed that these interventions significantly improved patient motivation and compliance with the recommended treatment regimen. Further, the data showed that this regimen, which consists of good hygiene, elevation of the leg, exercise, bandaging, and treatment and prevention of entry lesions with topical antifungal and antibacterial creams, can be done at home and be integrated into the daily activities of the affected women (41). Formative research techniques, including focus groups and personal interviews, are being used 1) to determine the feasibility, effectiveness, and cost of an elephantiasis treatment program in a community setting in Haiti; 2) to understand the economic, health, and social impact of the disease in Haitian women; 3)

to develop public health messages that will enhance the effectiveness of the treatment program and evaluate patient compliance with the program; and 4) to understand the attitudes and practices of Haitian medical providers regarding disease treatment and develop educational programs for medical providers.

### Conclusions

Communication theory and techniques, aided by the electronic revolution, provide new opportunities and challenges for the effective transfer of laboratory, epidemiologic, surveillance, and other public health data to the public who funds them. In what has been called the information age, health data are increasingly demystified by the communication media and are claiming their place in the marketplace as a public commodity. With health information readily available, the final decisions (and responsibility) about individual health are increasingly transferred from the health-care provider to the patient who is most profoundly and directly affected by treatment and prevention measures.

### Acknowledgment

The authors thank Carole Craft for her editorial suggestions and support.

Dr. Freimuth is Associate Director for Communication at the Centers for Disease Control and Prevention, Atlanta, Georgia. Her research focuses on the role of communication in health promotion.

### References

1. Freimuth V, Cole G, Kirby S. Issues in evaluating mass mediated health communication campaigns. In: WHO monograph "Evaluation in health promotion: principles and perspectives." Copenhagen: WHO Regional Office for Europe. In press 2000.
2. Prochaska JO, Diclemente CC, Norcross JC. In search of how people change: applications to additive behavior. Am Psychol 1992;47:1102-14.
3. Freimuth V. Developing the public service advertisement for nonprofit marketing. In: Belk RW, editor. Advances in nonprofit marketing: a research manual. Vol 1. New York: JAI Press Inc.; 1985. p. 55-93.
4. Becker MH, Haefner D, Kasl SV. Selected psychosocial models and correlates of individual health-related behaviors. Med Care 1977;Suppl:27-46.
5. Bandura A. Social foundations of thought and action. Englewood Cliffs (NJ): Prentice-Hall; 1986.
6. Rogers EM. Diffusion of innovations. 3rd ed. New York: The Free Press; 1983.
7. Gordon D. Therapeutic metaphors: helping others through the looking glass. Cupertino (CA): Meta; 1978.
8. Potter P. Using cultural metaphors to deliver effective prevention messages. The American Journal of Health Communications 1996;4-8.
9. Littman SK. Forward. In: Barker P, editor. Using metaphors in psychotherapy. New York: Brunner/ Mazel; 1985. p. vii-viii.
10. Zuniga ME. Using metaphors in therapy: dichos and Latino clients. Soc Work 1992;37:55-9.
11. Barden LS, Dowell SF, Schwartz B, Lackey C. Current attitudes regarding use of antimicrobial agents: results from physicians' and parents' focus group discussions. Clin Pediatr 1998;665.
12. Heersink M. *E. coli* O157: the true story of a mother's battle with a killer microbe. New Jersey: New Horizon; 1996.
13. Miller MH. Seeking advice for cancer symptoms. Am J Public Health 1973;63:955-61.
14. Woods DR, Davis D, Westover B. America Responds to AIDS: its content, development process and outcome. Public Health Rep 1991;106:616-22.
15. Rogers EM, Story JD. Communication campaigns. In: Berger C, Chaffe S, editors.. Handbook of communication science. Newburg Park (CA): Sage Publication; 1987.
16. Brown JD, Childers KW, Waszak SC. Television and adolescent sexuality. Journal of Adolescent Health Care 1990;11:62-70.
17. Gerbner G, Gross L, Murgan M, Signiorelli N. Living with television; the dynamics of the cultivation process. In: Bryant J, Zillmann D, editors. Perspectives in media effects. Hillsdale (NJ): Lawrence Erlbaum Associates; 1986.
18. Freimuth VS, Stein JA, Kean TJ. Searching for health information: the cancer information service model. Philadelphia: University of Pennsylvania Press; 1989.
19. Alcaley R. The impact of mass communication campaigns in the health field. Soc Sci Med 1983;17:87-94.
20. Gandy OH. Beyond agenda setting. Norwood (NJ): Alblex; 1982.
21. Klapper J. The effects of mass communication. Glencoe (IL): Free Press; 1960.
22. McCombs ME, Shaw DL. The agenda-setting function of the media. Public Opinion Quarterly 1972;36:176-88.
23. Wallack L. Mass media and health promotion: promise, problem, and challenge. In: Atkin C, Wallack L, editors. Mass communication and public health. Newbury Park (CA): Sage Publication; 1990.
24. Scott SA, Jorgensen CM, Suarez L. Concerns and dilemma of Hispanic AIDS information seekers: Spanish-speaking callers to the CDC national AIDS hotline. Health Educ Behav 1998;25:501-16.
25. Sonnenberg A. Peptic ulcer. In: Everhart JE, editor. Digestive diseases in the United States: epidemiology and impact. Washington: U.S. Department of Health and Human Services, Public Health Service, National Institutes of Health, 1994:359-408; NIH publication no.94-1447.
26. American Digestive Health Foundation and Opinion Research Corporation. Familiarity with *H. Pylori* among adults with digestive disorders and their views toward diagnostic and treatment options. Bethesda (MD): American Digestive Health Foundation and Opinion Research Corporation; 1995.
27. Winik LW. The virus hunters. Parade 1998;Feb 8:6-9.
28. Kennedy M. The killer germ. Time 1998;Aug 3:56-62.

29. Centers for Disease Control and Prevention. Hepatitis A associated with consumption of frozen strawberries—Michigan, March 1997. MMWR Morb Mortal Wkly Rep 1997;46:288,295.
30. Khan AS, Tshioko K, Heymann DL, Le Guenno B, Nabeth P, Kerstiens B, et al. The reemergence of Ebola hemorrhagic fever, Democratic Republic of the Congo, 1995. J Infect Dis 1999;179 Suppl 1:S76-86.
31. Bandura A. Analysis of modeling processes. School Psychology Digest 1975;4:4-10.
32. Center for Communication Programs, The Johns Hopkins University, The Annenberg School for Communication, University of Southern California, and Center for Population Options. Proceedings from the enter-educate conference: entertainment for social change. April 1989.
33. Center for Communication Programs, The Johns Hopkins University. Reaching young people worldwide: lessons learned from communications projects. 1986-1995. Working page No. 2, October 1995.
34. Wallack L, Dorfman L, Jernigan D, Thomba M. Media advocacy and public health: power for prevention. Newbury Park (CA): Saga Publications; 1993.
35. Osterholm MT, Potter ME. Irradiation pasteurization of solid foods: taking food safety to the next level. Emerg Infect Dis 1997;3:575-7.
36. Centers for Disease Control and Prevention. Addressing emerging infectious diseases threats: a prevention strategy for the United States. Atlanta: U.S. Department of Health and Human Services, Public Health Service; 1994.
37. Toner M. When bugs fight back. The Atlanta Journal and Constitution 1993 Aug 23, Oct 16.
38. Graham G. Meeting the public's need for information during the hantavirus outbreak. Am J Public Health 1996;86:8.
39. Kreeger K. One year later, the hantavirus investigation continues. The Scientist 1994;8:14.
40. Eberhard ML, Walker EM, Addiss DG, Lammie PJ. A survey of knowledge, attitudes, and perceptions of lymphatic filariasis, elephantiasis, and hydrocele, among residents in an endemic area in Haiti. Am J Trop Med Hyg 1996;54:299-303.
41. Coreil J, Mayard G, Louis-Charles J, Addiss, D. Filarial elephantiasis among Haitian women: social context and behavioral factors in treatment. Trop Med Int Health 1998;3:467-73.
